# Information flow as reproductive governance. Patient journey analysis of information barriers and facilitators to abortion care in the republic of Ireland

**DOI:** 10.1016/j.ssmph.2022.101132

**Published:** 2022-05-19

**Authors:** Deirdre Duffy, Joanna Mishtal, Lorraine Grimes, Mark Murphy, Karli Reeves, Dyuti Chakravarty, Wendy Chavkin, Mary Favier, Patricia Horgan, Bianca Stifani, Antonella F. Lavelanet

**Affiliations:** a Department of Social Work and Social Care, Manchester Metropolitan University, Manchester, United Kingdom; b Department of Anthropology, University of Central Florida, Orlando, FL, USA; c Social Science Institute, Maynooth University, Republic of Ireland; d Eldon Family Practice, Dublin, Republic of Ireland; e School of Sociology, University College Dublin, Republic of Ireland; f Global Doctors for Choice, New York, NY, USA; g Parklands Surgery, Cork, Republic of Ireland; h Broad Lane Family Practice, Cork, Republic of Ireland; i Department of Obstetrics, Gynecology and Women’s Health, Montefiore Medical Center, New York, USA; j UNDP-UNFPA-UNICEF-WHO-World Bank Special Programme of Research, Development and Research Training in Human Reproduction (HRP), Department of Sexual and Reproductive Health and Research, World Health Organization, 20 Avenue Appia, 1211, Geneva, Switzerland

**Keywords:** Abortion access, Reproductive governance, Information flow, Barriers, Facilitators, Ireland

## Abstract

**Background:**

Information flow – information communication and transmission pathways and practices within healthcare systems – impacts patient journeys. Historically, regulating information flow was a key technology of reproductive governance in the Republic of Ireland. Pre-2018, law and the State sustained informational barriers to and through abortion care in Ireland. An expanded abortion service was implemented in January 2019.

**Method:**

Patient Journey Analysis (PJA) interrogates informational facilitators and barriers to/through post-2019 abortion care in Ireland. We focus on information flow at the interfaces between the ‘public’ sphere and ‘point of entry’, ‘point of entry’ and primary care, and primary and secondary care.

**Materials:**

The paper uses data from a mixed-method study. A tool for assessing online abortion service information (ASIAT), desktop research, and qualitative data from 108 in-depth interviews with providers, policy-makers, advocacy groups, and service users informed the analysis.

**Results:**

Abortion patient journeys vary. Information flow issues, e.g. communication of how to access services, referral systems, and information handover, act as barriers and facilitators. Barriers increase where movement from primary to secondary is needed.

**Applications:**

The article identifies good practice in information flow strategy, as well as areas for development. It illustrates the significance of information flow in accomplishing reproductive governance.

## Highlights

• Information flow facilitating abortion access was restricted in Ireland under pre-2018 legislation.
• Information flow can now act as a barrier and facilitator to patient journeys to abortion care.
• The ‘informational barriers/facilitators’ vary as the patient journey progresses.
• Pro-active promotion and targeting disinformation address informational barriers at ‘point of entry’.
• Referral mechanisms and information handover systems can cause delays as patient journeys progress.

## 1 Introduction

After a 2018 popular referendum, the Republic of Ireland (hereafter Ireland) expanded abortion access under new legislation – the Health [Regulation of Termination of Pregnancy] Act 2018, hereafter the Health Act 2018 (Mullally et al., 2020b). From January 1^st^ 2019 Ireland implemented a ‘whole system’ reform of the delivery of abortion care. The Act outlined four grounds for abortion (Donnelly & Murray, 2020): risk to maternal life or health (prior to fetal viability) (Section 9); immediate risk to life or of serious harm (Section 10); condition likely to lead to death of the foetus before, or within 28 days of, birth (Section 11); and where the pregnancy is under 12-weeks gestation (dated from the first day of the last menstrual period) (Section 12) (see Table 1).

**Table 1:** Qualitative sample characteristics.

Sample label and count	Sub-sample labels and count	Notes	Recruitment method
Providers **n = 51**	Community providers **(n = 23)** Hospital providers **(n = 28)**	Community providers worked in 12 of 26 counties Hospital providers worked in maternity hospitals in 9 of 26 counties	Announcement mailout to the Irish College of General Practitioners (ICGP) membership. WhatsApp network (facilitated by local advisors)
Key Informants **n = 27**	Policy making and contract negotiation **(n=2)** HSE implementation and service facilitation **(n=16)** Advocacy (domestic and international) **(n=14)**	1 participant worked in policy making and HSE implementation 4 participants worked in service facilitation and advocacy	Direct contact with relevant HSE offices and stakeholder organisations.
Service Users **n = 30**	Medical Abortion **(n=24)** Hospital-based care for foetal anomaly **(n=6)**	Under 18-year-olds excluded Age range 23 – 47 years old All completed second-level/high school Income range €190-€2,200 per week No participants identified as trans or non-binary	Open advertisement on three social media platforms (Twitter, Facebook, and Instagram). Twenty-three GP offices across Ireland also distributed study flyers.

Care is provided through Ireland’s public health service, the Health Service Executive (HSE), free of charge. A ‘community pathway’ for medical abortion (MA) up to 9-weeks and 6-days/69-days gestation (9 ​+ ​6 ​GA) delivered by primary care providers in general practice (GPs) and Family Planning Clinics.^1^ Over 10 weeks, care is delivered via integrated pathways involving primary and secondary care collaboration. Dating scans care be requested to confirm GA. On the Institute of Obstetricians and Gynaecologists’ recommendation, the HSE contracted a private agency for gestational dating scans for patients seeking abortion care. Most abortions since 2019 have been performed under Section 12, under 12-weeks gestation (Department of Health, 2020; 2021).

Sections 9-11 require certification by two medics. Care under Sections 9 and 10 must be led by a consultant obstetrician, with the support of a multi-disciplinary team (MDT). Patients eligible under Sections 9 (risk to life or serious harm to health) and 10 (immediate risk to life or serious harm) of the legislation can access secondary care directly (Mullally et al., 2020a). Care is integrated and involves input from different specialists. Regardless of the section on the Act the initial application falls under, if the pregnancy is under 9-weeks and 6-days GA, the MDT may redirect patients to the ‘community pathway’.

Theoretically, the 2018 legal reforms enable the exercise of greater reproductive agency. However, following Morgan and Roberts’ (2012) reproductive governance framework, reproductive freedoms are liberalised and stymied by a combination of factors including and beyond legal changes. The economics of abortion (Coast et al., 2018), training for health care workers, and the availability of clinics (Sethna & Doull, 2013) have all been used as mechanisms for “different historical configuration of actors – such as state, religious, and international financial institutions, NGOs, and social movements – […] to produce, monitor, and control reproductive behaviours and population practices” (Morgan & Roberts, 2012, p. 243). These configurations are entangled with and reinforce moral regimes of abortion (Moore et al., 2021).

Through reproductive governance, social science scholars interrogate how the objectives of different actors are accomplished. Morgan (2019) notes that the main technologies discussed in the literature are judicial. However, the organisation and practices of health systems are central technologies of reproductive governance. Suh (2018, 2020) identifies how patient data collection in post-abortion care influence reproductive autonomy. In Suh’s work, the Senegalese governments’ emphasis on reporting PAC as connected to miscarriage of desired pregnancies rather than necessary for desired abortions, has led to valorisation of and investment in programmes for contraception and safe maternal health. How PAC information is recorded thus impacts service investment and availability and health care workers’ attitudes. These issues are known to facilitate or impede abortion care trajectories (Coast et al., 2018; Coast & Murray, 2016; Fink et al., 2016). Furthermore, Suh and others contend that informational practices have not only undermined provision, but left room for moral judgements about ‘good’ and ‘bad’ abortions to flourish(Haaland et al., 2020; Suh, 2018).

We propose 'information flow’ as a further technology for accomplishing reproductive governance. Information flow research examines how the communication, processing, and transmission of information to and through integrated health care systems shapes patient progress (Hoonakker et al., 2019; Kneck et al., 2019). Information flow recognises that communication pathways and practices between providers impact access to care(Manias et al., 2016).

We interrogate how information flow both between the health service and the public and between healthcare actors and institutions involved in the delivery of care facilitate or disrupt patient journeys to abortion. We focus specifically on facilitators and barriers at the interfaces between the public domain and point of entry to abortion care, point of entry to primary care, and primary care and secondary care and adopt a user-centric approach – patient journey analysis. We argue that information flow at these points stratifies abortion access. This resonates with recent discussions on abortion service information (Dodge, 2017; Kavanaugh et al., 2019), provider communication (Dianat et al., 2020), information referral between services (Zurek & O'Donnell, 2019; 2014) and information governance(Zanini et al., 2021).

The paper is based on a World Health Organisation commissioned study of facilitators and barriers to abortion care in Ireland. The research was mixed-method and combined macro (i.e. national service designers’ policy-makers’ accounts, government reports), meso (i.e. practitioner and advocacy groups’ accounts, practitioner guidance), and micro (i.e. service user accounts, online information) data.

The paper is structured in three parts. First, we outline how information flow has been used in Ireland to control and facilitate abortion access. Second, we present a patient-centric analysis of informational facilitators and barriers in abortion care following the 2018 reforms. Third, drawing on reproductive governance we discuss the contingencies of the liberalisation of abortion access in Ireland and consider how information flow accomplishes control over reproductive behaviours and subjects.

## 2 Reproductive governance and information flow

Historically, in Ireland, information flow for abortion has been as a key technology for limiting abortion access. The 1995 Information Act expressly prohibited open public-facing communications about abortion services by health providers in Ireland, including specialist family planning clinics (FPCs) (Carder, 1996; Koegler, 1996). Referral for abortion care was restricted and information systems could not facilitate patient progress to abortion directly. Out of concern for continuity of care and patient safety, some providers established their own information handover processes for patients who decided to access abortion care outside the State(Duffy et al., 2018). These were ad hoc and there was no clinical guidance framework for information handover in abortion care. Pro-choice organisations, activist groups, and providers outside Ireland addressed some of the informational barriers to care(Duffy, 2020). This combination of factors impeded abortion access and worked together to control reproductive behaviours on the island of Ireland (Morgan & Roberts, 2012). Abortion seekers frequently travelled for services or used illegally-imported abortion pills (Bloomer et al., 2018).

Following the 2018 referendum, the Department of Health transformed the framework for information flow in abortion care entirely. Information flow was reoriented towards a model of care which would, in principle, facilitate access to abortion. The reforms included the formation and public promotion of a state-run abortion helpline service (MyOptions). Unlike the HSE’s previous crisis pregnancy counselling service, MyOptions was explicitly established as an abortion information service. Although non-directive, MyOptions main function is to provide contact details of registered abortion providers in primary care.^2^ The service is not an ‘appointments booking service’.

Additionally, the Irish College of Obstetrics and Gynaecology, Irish Nursing and Midwifery Organisation, and Irish College of General Practitioners produced clinical guidance related to the expanded abortion care pathways. Guidance for the ‘community pathway’/care pathway for termination of pregnancy under 12 weeks gestation (section 12 of the new legislation) was produced in November 2018. Guidance for termination of pregnancy over 12 weeks gestation (sections 9 to 11) was produced in early 2019. This guidance outlined referral processes including what information would be required at handover from primary to secondary care.

The professional regulations on information provision between health care professionals and patients were revised with specific attention to information flow. The revised Code of Conduct for Professional Ethics (Irish Medical Council, 2019) compels health care professionals to provide sufficient information to patients to facilitate access to abortion care regardless of personal beliefs. This includes details of the MyOptions service and the HSE’s patient guides (*Your Guide to Medical Abortion*). Unlike the Information Act 1995, the revised IMC Code emphasises respecting patient’s decisions rather than avoiding making abortion ‘attractive’.

From a reproductive governance perspective, this combination of judicial, policy-level, and professional interventions should work together to facilitate abortion access through transforming information flow. However, before we can reach this conclusion, it is important to explore the patient journey to see if and how other factors work against this liberalisation, for whom, and under what circumstances.

## 3 Methods

The article adopts Patient Journey Analysis, a methodology used in health research to identify what shapes a patient’s progress through a health care system (Percival & McGregor, 2016). PJA is patient-centric in that it is concerned with what impacts patient progress. It is multidimensional, drawing together macro-, meso-, and micro-level processes and outcomes rather than solely focusing on micro-level decision-making or how individuals navigate and experience the lifeworlds of healthcare. PJA (McCarthy et al., 2016) focuses on what makes patient progress to and through health care more or less seamless, with the aim of identifying and minimising fragmentation, variation, and inequities.

PJA involves process tracing of patient journeys using data from a range of sources including secondary data, primary data from providers and service managers, health care activists and advocacy groups, and patients themselves. Specifically, it pays attention to how the individual moves from one point of a health care journey to another (i.e. from home to primary care provider, from primary care to secondary care and so on) and the interaction between individuals and service providers (Carayon et al., 2020). This concern with transition points or interfaces between different points on health care journeys resonates with wider sociological research on interactions between health care professionals in different settings and how these shape patient journeys. Spendlove (2018) addresses this as ‘boundary work’. Yet, unlike other methodologies (e.g. pathway analysis, trajectory analysis), PJA argues that patient journeys begin outside health care organisations (McCarthy et al., 2016) and thus explores the interface between the ‘public sphere’ and ‘point of entry’ to health systems as well as interfaces within health care services.

## 4 Materials

Our PJA used document data, quantitative data, web-based information objects, and qualitative data from interviews. Document data included the Irish Medical Council’s (IMC) *Guide to Professional Conduct and Ethics for Registered Medical Practitioners (Amended) 2019* and core guidance on termination of pregnancy under the new legislation produced by the key professional organisations for Irish health workers involved in abortion care – the Institute of Obstetricians and Gynaecologists (IOG) and the Irish College of General Practitioners (ICGP) – in 2018 and 2019. We also used the two patient-facing documents produced by the HSE, including *Your Guide to Medical Abortion* and *Your Guide to Surgical Abortion* and web-based information on accessing abortion services on HSE webpages.

Quantitative data included abortion statistics in the public domain accessed from the Irish Department of Health (DOH), the UK Department of Health and Social Care (DHSC), and the Netherlands’ Inspectorate of Healthcare and Youth. The Health Products Regulatory Authority’s (HPRA) Enforcement Section (Compliance Office), the Abortion Support Network (ASN), and Women Help Women (WHW) also provided anonymised secondary data.

We used a method – the Abortion Service Information Appraisal Tool (ASIAT) – to gather web-based information objects. ASIAT was developed by the lead author in a previous study to mimic online health service information seeking behaviours; which is based on pre-existing implements for assessing online health information seeking (Best et al., 2013; Duffy et al., 2019; Liu, 2005; Sillence et al., 2007). As part of the ASIAT process, we searched for abortion service information webpages on two popular search engines (Google and Bing) using eight search terms in July 2020 from an Irish IP address. One researcher (LG) led the retrieval of webpages at this stage and collated results, including dated screenshots and URLs. The search terms were: “where can I get an abortion”; “where can I get an abortion Ireland”; “how can I get an abortion”; “how can I get an abortion Ireland”; “getting an abortion”; “getting an abortion Ireland”; “I need an abortion”; and “I need an abortion Ireland”. A total of 374 webpages were returned. The lead author then removed broken, duplicate links and webpages which did not contain any information on access to abortion services in Ireland (i.e. webpages discussing abortion or the referendum in Ireland more generally). This left 71 webpages.

Finally, PJA used qualitative data coded as relating to information and information flow gathered as part of the larger implementation study. In addition to the ASIAT and desktop analysis, the implementation study included in-depth interviews (IDIs) with service users, providers, and key informants from advocacy organisations, the HSE, and the Department of Health. The total IDI sample was 108, with all interviews undertaken between May 2020 and March 2021. The sample and sub-sample characteristics are outlined in brief in the Table 1 below.

The multi-step coding process of qualitative data included open and axial coding, and followed the “dynamic and fluid process” of the grounded theory approach(Corbin & Strauss, 2015). The qualitative methodology is explained in full in peer-reviewed publications related to the study (Mishtal et al., 2022).

The study’s methodology, findings, and recommendations underwent expert checking via consultations with Irish team members who acted as local advisers in Dublin and Cork and an external adviser to the study in Cork, Ireland.

This study received ethical approvals by the World Health Organization Ethics Review Committee (ID# A66001, 24 April 2020), and the University of Central Florida Institutional Review Board (ID# STUDY00000846). A written informed consent was obtained from key informants and healthcare providers, and a verbal informed consent was obtained from service users.

## 5 Results and analysis

### 5.1 Public sphere to ‘point of entry’ interface

Data indicate limited informational barriers to ‘point of entry’, in relation to abortions provided under Section 12 of the Act. ASIAT analysis indicates that the ‘point of entry’ for abortion services under Section 12 is prominent online. Over half of webpages (n = 45) relating to abortion access in Ireland returned through searches were owned by the HSE and contained a webchat function for contacting MyOptions. Documents produced for health professionals and patients also emphasise MyOptions as the key ‘point of entry’.

The precise ‘point of entry’ for abortion services under Sections 9–11 is less clear; no webpages returned through searches outlined the ‘point of entry’ for care under these grounds.

The HSE led a large-scale promotion campaign in advance of and during the roll out of services. From November 2018 to February 2019 media campaigns and advertising through social, radio and news media promoted MyOptions and abortion services. There was a widespread advertising campaign with posters on *“the bus shelters, the Luas [the tram service in Dublin], the back of toilet doors in some premises”* (KI17*); “college washrooms or in pubs or clubs, like the back doors of the washrooms or, you know, at the sink facilities as well”* and *“leaflets were sent to GP surgeries and pharmacies as well. And posters.”* (KI8,9)

While some health providers and service users observed that the active promotion of ‘point of entry’ has waned since the initial service launch - as one provider remarked, *“it seems to have gone off the radar”* (Provider 18) - most service users interviewed were aware of how and where to access care. Data indicate that the combination of public advertisements online in the media, personal networks and the broader discussion of abortion heightened awareness of the availability of abortion services:

“I knew the My Options helpline existed so I Googled it. I don't know how I know. I guess it is just common knowledge at this stage.” (Service User 8)

“After I found out that I was pregnant, I looked up online for options. That’s where I learned about the My Options helpline.” (Service User 9)

“A friend of mine had used the My Options helpline. She told me to ring them even if for a chat because I wasn't sure what I wanted at that stage.” (Service User 11)

Providers said they had *“not been aware that there was a difficulty with getting information to women.”* (Provider 7) and that their patients were usually *“very well-informed”* (Provider 20). Our PJA indicates that the available information facilitates individuals’ movement to ‘point of entry’ to care.

Yet PJA suggests inequalities in informational facilitators and barriers at ‘point of entry’. This is consistent with research on patient journeys which highlights the differential experience of various communities (Carayon et al., 2020). For example, disinformation is not a substantial barrier for non-migrant communities in Ireland. ASIAT indicates a limited circulation of disinformation; searches did not retrieve any disinformation webpages. Key informants described how disinformation was “really proactively managed” (KIs 3 and 4), with ‘hoax’ websites pursued through the courts in 2019. Furthermore, service users stated that they were confident in their ability to identify and avoid disinformation:

“From stuff mentioned on Facebook and Twitter, I know that there are rogue crisis pregnancy agencies that are trying to mislead women. I know that there are pro-life leaflets being distributed outside GP practices” (Service User 4)

“I was lucky enough to not come across misleading information. But I would have known if something wasn’t right because of my investment on the topic” (Service User 21)

Yet encounters with disinformation were reported by respondents in relation to migrants. This is illustrated in comments such as the below account from a GP:

“And the other lady who I saw recently, she rang a counselling service that she found online when she discovered she was pregnant. And I think she wanted … Anyway, this was a service that was masquerading as providing information and in fact they told her yes, we can arrange a scan for you, but it’s going to take two weeks. ” (Provider 18, GP)

PJA indicates that lack of knowledge or understanding about how to access abortion services obstructs already marginalised abortion seekers’ movement towards ‘point of entry’; this includes service users living in rural areas. Rural respondents argued that ‘point of entry’ to abortion *“wasn’t openly promoted”* (Service User 10) and they *“didn’t come across public announcements about the policy after Repeal”* (Service User 21) where they lived. Key informants involved in advocacy raised concerns in relation to migrants:

“[Asylum seekers] don’t know how to access anything. They know that, you know, or actually a lot of people [know] thought abortion was legal in Ireland before it was, but even afterwards, they don’t know how to access it. Right? They don’t know how to get a GP. They can’t go to a GP unless they have a medical card, but the Reception and Integration Agency [the agency managing asylum applications] is not giving them the medical care because they don’t have a PPS number.” (Key Informant 19)

PJA suggests that the reasons for accessing abortion services is potentially relevant. ‘Point of entry’ for abortion seekers under Sections 9–11 and for abortion over 83-days is less visible. Although ASIAT analysis retrieved pages referencing the availability of abortion care over 12-weeks gestation in Ireland, no webpages outlined how or where to access care under these sections specifically, advising abortion seekers to speak to MyOptions.

Overall the PJA indicates that, while the HSE’s information strategy to make ‘point of entry’ for Section 12 visible for the general population has been successful, migrants and rural populations remain vulnerable to informational barriers. Additionally, the ‘point of entry’ for Sections 9 -11 is less obvious. Public advertising does not discuss where to access abortion under these grounds.

### 5.2 ‘Point of entry’ to primary care interface

Qualitative data show that the service users who did not contact MyOptions experienced less direct patient journeys (Carayon et al., 2020). Service users who first approached GPs (i.e. without going through MyOptions) relayed problems with non-referral, despite the clarification of the obligation to refer for abortion services in a timely fashion by the IMC 2019 *Code*. These problems ranged from a failure to provide clear, precise information to assist the movement of the patient to abortion care, to dissuasion:

“I was shocked by my GP. She was trying to be nice but was trying to talk me out of it. If you aren’t providing, then you should at least give information about other providers. She only told me about IFPA. She didn’t provide me with any other numbers.” (Service User 15)

Provider qualitative data suggest that non-referral may be related to lack of engagement with the service during the initial implementation:

“I think a lot of GPs don’t have even the information to say, you know, if somebody wanted to ring up a GP, they would get through to the receptionist there, but unless that they’re involved in the service or have been from the start, they may not know about, you know, either the My Options help line or any other GP that they’re aware of doing it.” (Provider 38, Health Centre manager)

FPCs in Ireland have registered with the HSE to provide abortion services at some of their clinics. However, qualitative data indicate that approaching FPCs directly sometimes delayed patient journeys. When the clinics did not have capacity to provide abortion care themselves, they directed abortion seekers back to MyOptions:

“I rang [FPC]. They said they wouldn’t have an appointment available for another 10 days. I didn’t want to wait that long so she gave me the number of the My Options helpline.” (Service User 20)

Overall, PJA indicates that the key informational facilitator to primary care is MyOptions and the main informational obstructor is non-referral. Furthermore, while FPCs registered as primary care providers were consistently reported as providing direct, clear referral information to MyOptions, from a PJA perspective this represents a partial obstructor as it delayed patient journeys.

### 5.3 Primary to secondary care interface

Abortion seekers from Ireland over 12-weeks gestation are still accessing abortion services in other jurisdictions. The UK Department of Health and Social Care’s (DoHSC) annual abortion statistics bulletin reported 277 patients over 12-weeks gestation providing Irish addresses at abortion clinics in England and Wales in 2019 (Care, 2020) and 176 in 2020 (Care, 2021). These data show only a limited decline in abortions which could fall under Sections 9–11 of the new legislation. In 2019, 64 Irish residents accessed abortions under Ground E of the UK Abortion Act 1967 (foetal anomaly; equivalent to Section 11 of the Irish law) and 63 in 2020. This compares with 84 reports in 2018 prior to the new legislation.

Data provided by the Abortion Support Network (ASN), who provide financially support to people travelling from Ireland for abortion care, also indicate that while abortion travel has dropped overall, people still travel from Ireland for abortion over 12-weeks gestation. While ASN supported more than twice as many Irish residents in 2018 than in 2019 and 2020 combined (2018 n = 625; 2019 and 2020 = 302), the monthly average for Irish residents over 12-weeks gestation supported by ASN was between 7 and 8 people in 2018, 2019, and 2020.

Data from service users whose patient journey progresses to secondary care in Ireland describe the patient journey in largely positive terms. However some respondents found the movement to secondary care complicated and confusing:

“I'm a very organised person. I know the system. My background is in nursing. I was shocked by how confused I was by everything.” (Service User 10)

PJA indicate that professional networks are key facilitators at the primary/secondary care interface. Providers stated that these networks clarified who primary care providers should contact to facilitate service user transition to secondary care as well as ‘in-hospital’ processes and information handling systems. These networks were not established in the clinical guidance documents produced by either the HSE or professional organisations such as the IOG and ICGP prior to implementation but by providers themselves:

“We established a relationship and then [the GPs] have regular meetings and usually myself or [colleague], we are invited to those meetings to keep up to date with what’s happening. [ …. ] There are a lot of GPs referring from [county 1] to us because their hospital is not providing services at all. And then [hospital in county 2], it’s providing only for fatal foetal abnormality, so less than 12 weeks will come either to us or to [hospital in county 3]. So we are in touch with the GPs from those areas as well.” (Provider 42, OBGYN)

“The consultant invited in the GPs also to talk to them about that so that there was clear kind of pathways for the women from the community into the hospital and then I suppose navigating and really getting clear about the guidelines and the parameters of like diagnosis and waiting period, three days, and then you know, I suppose just navigating all of that.” (Provider 33, Nurse)

Data show variation in the format of information and the professional background of the people who co-ordinated information handover between primary and secondary care. As the interviewees quoted below described, both of whom are based in different hospitals, information is passed through a range of formats (electronically, by fax, and verbally) to clinicians and non-clinicians:

“So basically the pathway between community and the hospital here in [county] is that there is a specific contact number held by what we call the ambulatory gynaecology nurse or midwife […]. So the GP will ring this number, fax a referral. So you know, if it’s over nine weeks or if somebody’s less that nine weeks, but requires a scan for whatever clinical indication, it all comes through this and through the gynaecology midwife number.” (Provider 32, Midwife)

“[Commenting on practicalities of referral pathway] It can be an email, it can be a phone call […]. And then it all goes, there’s a dedicated secretarial staff and then there’s dedicated midwives for that clinic. So they just process the referrals, the same way as the referrals would be processed for any other clinic in the hospital, just with more urgency.” (Provider 24, OBGYN)

Hospital-based respondents stated that these information systems facilitated patient journeys for abortion under Sections 11 and 12. However, primary care providers gave a very different perspective. GP interviewees argued that the variation of who was involved in information handover could obstruct transitions. For example, some hospitals sub-contracted a private sector company to perform gestational dating scans. According to some primary care providers, this company *“… [is] not easy to deal with”* (Provider 20, GP) and, as hospitals do not always provide scans for abortion seekers, this leads to *“another two or three days’ delay”* (Provider 20, GP). The absence of information at handover could obstruct timely patient journeys, even where a health provider had identified a potential risk to health or serious harm justifying a Section 9 or 10 application. If a referral of a service user is made for *"the first time they are seen in [hospital 1]. Their GP has written in very concerned this person is suicidal"* but the patient arrives without a scan, the psychiatry team can offer them an appointment, but the lack of a scan will delay the patient (Provider 45).

Assigning non-clinical staff to co-ordinate referral and information handover is also potentially obstructive. Although hospital providers argued that non-clinical staff who worked in their teams *“every day […] for a couple of years”* (Provider 24, OBGYN) were sufficiently experienced and familiar with hospital systems to ensure information was transferred correctly, some noted that there may be delays if clinical questions were raised by GPs. As one OBGYN explained in relation to queries about whether a patient could be supported through the community pathway or not:

“When it’s a clinical person […] they’re able to probably give […] feedback [on queries] quite quickly to the GPs whereas in our system, because it’s an administrator, those queries have to get, you know, emailed in […] a group email to myself and to a few of the other people involved in the service […] So that can take a little bit of time because obviously it depends on how quickly you look at emails and reply and if someone’s on leave*.”* (Provider 31, OBGYN)

PJA suggests information format is an issue. GPs problematised the use of paper-based information in referral, arguing that it impeded post-abortion care as they were not able to ‘follow’ patient journeys through the abortion care system. This is outlined in the following response from a GP:

“But you know, it would be nice to know what exactly has happened because sometimes as well […] we realised [the patient needed] a hospital appointment and then they come back to us to talk about contraception afterwards. But we never know what’s gone on in the hospital. So if a patient comes back to us then to talk about contraception, we have to go through their whole details with them because we don’t have anything from the hospital.” (Provider 28, GP)

PJA points to the number of staff involved in co-ordinating transition as challenging. Data from this implementation study and other analyses of abortion services in Ireland(O'Shaughnessy et al., 2021; Power et al., 2021) indicate that individual or small teams of staff ‘championing’ abortion care often assume responsibility for ensuring all patient information is passed from primary to secondary care at handover. This means that patient journeys are vulnerable when staff familiar with requirements are unavailable, as outlined by one hospital-based provider:

“[The usual co-ordinator] was on leave there for two weeks in August and oh my God, it was such a nightmare because like there was somebody assigned to fill in for her, but they really didn’t know. They hadn’t been trained properly. They don’t know exactly what to do, so when I went to see somebody, look up the charts at clinic that evening, there was nothing in the charts. So I didn’t know why this patient was coming. I didn’t have certification. I didn’t know was she pre- or post-EMA or you know, what was going on. So just none of the information from the GP correspondence had been put in the chart and just, you know, it was wasting time following that up.” (Provider 31, OBGYN)

Our PJA illustrates that patient journeys over 12-weeks gestation under Sections 9 (health and harm under threshold of viability) and 10 (immediate risk to health and harm) of the new legislation may be protracted due to information. IOG guidance states that an obstetric-led multidisciplinary team must certify care by confirming that an abortion would mitigate risks. While the IOG states that abortion could be recommended, as a respondent based in psychiatry notes, the guidance allows for varied interpretations:

“because [guidance on when abortion is appropriate] is not unobjective, it is so subjective, and we haven’t received any guidance from any professional body on how to make that slightly more objective. We are left with personal interpretation, and so one psychiatrist might view it one way, another might view it the other, and there is a lot of wiggle room in either direction.” (Provider 45)

These patient journeys require consensus between health professionals on MDTs convened on a case-by-case basis. This can take time, resources and co-ordination. However, as another HSE key informant argued, this approach is required to ensure appropriately tailored care for these complex cases:

“I think you need a sort of a pragmatic individualised approach to these women, I’m not sure a pathway necessarily solves that, because […] they need very individualised care, often involving several different specialists.” (KI 26)

PJA suggests that collaborative networks between primary and secondary care health professionals have been key informational facilitators. The main informational barriers are the use of paper-based information, the reliance on individual or small teams of staff to co-ordinate transfer, and the use of non-clinical staff to co-ordinate referral and handover. PJA indicates that referral and co-ordinating care for patient journeys via the secondary care-led Section 9 grounds is potentially problematic. Patients under Section 9 rely on teams using guidance allowing subjective interpretation.

## 6 Discussion

From desktop data on abortion access one could infer that abortion patient journeys under Section 12 have been established successfully. Yet, following reproductive governance, relying on this data alone is problematic. Sanitised data on rates of access can obscure access inequalities and whether health systems are working to stratify reproductive access. While the pathway for abortion seekers under 9-weeks’ gestation in the community using early medical abortion (EMA) may be straightforward, the journeys of those who do not meet these criteria may be much more complex. Applying reproductive governance, this potentially points to the formation of a stratified abortion care regime where more socially acceptable abortions (in this instance EMA under 9-weeks) are facilitated by health services while more complex cases remain inadequately supported(Almeling, 2015; Suh, 2018).

Our PJA allowed us to explore whether abortion information communication, transmission and processing systems – information flow systems - established as part of the 2018 reforms work to stratify abortion access or not. PJA indicates how information flow in Ireland facilitates abortion care journeys, under which circumstances, and with what limitations. From our analysis, three aspects stand out. The promotional campaign across multiple platforms has been a highly successful information facilitator; public awareness of ‘point of entry’ is, from our data, generally high. MyOptions also represents a dramatic improvement; while it does not make appointments directly the service is experienced by the majority of our interviewees as facilitating patient progress to abortion. The fact that individuals can access information about their abortion care needs, as opposed to facing previous efforts to promote continuation of pregnancy is significant. At a secondary care level, collaborative working between primary and secondary care providers have strengthened referral and information handover.

That said, there are information flow issues, reflected through informational barriers to care at the transition between primary and secondary care for example. Shortages of staff to manage handover of patient information as service users move between settings can delay or protract patient journeys. This can impact abortion seekers at 9–12 weeks’ gestation, who require hospital-based care. Handover is being co-ordinated by staff without clinical training even though research in healthcare management and quality recommends clinically-trained staff lead handover(Manias et al., 2016). The findings also show that already marginalised communities and those living in rural areas are vulnerable to informational barriers at ‘point of entry’ as promotional campaigns are less visible outside cities. Such differential experience resonates with existing literature on patient journey analysis (Carayon et al., 2020) and abortion care (Coast et al., 2018). These reported barriers can have substantial impact on abortion access equity. Under the current legislation, terminations accessed under Section 12 must be completed before 12-weeks gestation. Migrants and people living in rural areas are potentially at a disproportionate risk of ‘timing out’ of the community abortion care pathway.

The findings also indicate that there is potential for delays in providing care for patients seeking abortion over 12-weeks gestation due to risk to health or serious harm. There is scope for subjective interpretation of whether an abortion is an appropriate treatment. Certification requires agreement by a multi-disciplinary team, a process that can make patient journeys more protracted. Structural limitations to information flow on these pathways, such as the availability of staff or cohesiveness of systems for processing patient information, appear to have received less attention. Providers in some areas have developed their own networks.

The analysis highlights the complexity of reproductive governance post-liberalisation. The operation of health systems makes timely, cohesive abortion care journeys contingent on a range of social and practical factors. In the post-liberalisation context of abortion care in Ireland, the rationale for abortion, the circumstances and characteristics of patients, and the gestation of the pregnancy are linked with variations in how easily accessible abortion care is. Information flow can impact variation, with implications for who can and cannot take advantage of the liberalised abortion care system. For example, the cohesiveness of handover or referral, can shape experiences of those accessing abortion due to foetal anomaly as compared with those accessing early medical abortion.

There are learning points here for policy-makers. The data on uneven information flow to ‘point of entry’, i.e. the shortage of visible promotion in rural areas and reported experiences of disinformation by migrants, suggest targeted communication is required. Similarly, public information outlining ‘point of entry’ for care under Sections 9 and 10, 9 and 10 of the legislation needs to be expanded; explanations of pathways for these applications are limited. Further guidance and discussion on Section 9 applications may be necessary to ensure timely patient journeys.

## 7 Limitations

The study has several limitations. The web-based information collection did not include social media outlets. Document analysis of abortion care guidance only includes documents available in the public domain or by request from the HSE. We did not have access to data on number of abortion providers in the Republic of Ireland or the distribution of services across the country. The service user sample is not representative of all patient groups or potential experiences. The sample limits commentary on the differential effect of education or socio-economic status (SES) on abortion access in Ireland, despite established literature on educational and economic barriers to abortion in other jurisdictions (Amado et al., 2010; Sethna et al., 2013)

## 8 Conclusions

Overall, this analysis indicates that, while the reformed information flow systems are certainly more facilitative for abortion service patients than prior to 2018, progress is uneven. The cohesiveness of the patient journey to and through abortion care is contingent on the circumstances of abortion seekers. Those seeking abortion at an early gestation, under-9 weeks, in the community pathway, are more likely to encounter an information flow that facilitates access effectively and efficiently than those progress to care involves information flow between primary and secondary care. Such inequities are systemic; variations in information handover and referral processes are the result of how the borders of primary and secondary are organised and resourced at a local level. Our analysis suggests that embedding an integrated information flow system across all the care pathways has received less attention at a national level than establishing information flow systems to facilitate progress to ‘point of entry’ or community care level.

Through a reproductive governance lens, Ireland reflects a national context where reproductive agency is not equal. It also resonates with global discussions about the opportunities for moral claims about good and bad abortions to influence reproductive autonomy. Our integration of information flow and reproductive governance indicates that questions remain as to whether the reforms have addressed barriers to abortion care or created new mechanisms for inhibiting abortion access.

## Funding source

This study was funded by the UNDP-UNFPA-UNICEF-WHO-10.13039/100004421World Bank Special Progra mme of Research, Development and Research Training in Human Reproduction(HRP), Department of Sexual and Reproductive Health and Research, 10.13039/100004423World Health Organization (Study ID: A66001). The funding was awarded to JM. AFL is a staff member of the UNDP-UNFPA-UNICEF-WHO-10.13039/100004421World Bank Special Programme of Research, Development and Research Training in Human Reproduction (HRP), Department of Sexual and Reproductive Health and Research, 10.13039/100004423World Health Organization. Sponsor's website: https://www.who.int/reproductivehealth/hrp/en/

## Ethics statement

This study received ethical approvals by the World Health Organization Ethics Review Committee (ID# A66001, 24 April 2020), and the University of Central Florida Institutional Review Board (ID# STUDY00000846). A written informed consent was obtained from key informants and healthcare providers, and a verbal informed consent was obtained from service users.

Qualitative interview data are available within the manuscript. We are not able to make full transcripts publicly available because doing so would violate our promised commitment to the participants’ confidentiality. Research participants signed informed consents to participate in the study which stated that any data they provide will only be used for the purposes of this study and in accordance with applicable European Union General Data Protection Regulation law. Participants did not provide consent to have their transcripts made publicly available.

## Declaration of competing interest

There are no conflicts of interest.

## Footnotes

1 FPCs in Ireland sit outside the HSE; not all FPCs are registered as abortion providers.

2 Providers do not have to register their contact details with MyOptions.
